# Cardiovascular Events after Community-Acquired Pneumonia: A Global Perspective with Systematic Review and Meta-Analysis of Observational Studies

**DOI:** 10.3390/jcm9020414

**Published:** 2020-02-03

**Authors:** António Tralhão, Pedro Póvoa

**Affiliations:** 1 Polyvalent Intensive Care Unit, Hospital de São Francisco Xavier, Centro Hospitalar Lisboa Ocidental, Estrada do Forte do Alto do Duque, 1449-005 Lisbon, Portugal; atralhao@chlo.min-saude.pt; 2 Cardiology Department, Hospital de Santa Cruz, Centro Hospitalar Lisboa Ocidental, Avenida Professor Doutor Reinaldo dos Santos, 2790-134 Carnaxide, Portugal; 3 NOVA Medical School, CHRH, New University of Lisbon, 1069-056 Lisbon, Portugal; 4 Center for Clinical Epidemiology and Research Unit of Clinical Epidemiology, OUH Odense University Hospital, DK-5000 Odense C, Denmark

**Keywords:** pneumonia, community-acquired, cardiovascular complications, acute coronary syndromes, heart failure, arrhythmias, stroke

## Abstract

Acute cardiovascular disease after community-acquired pneumonia is a well-accepted complication for which definitive treatment strategies are lacking. These complications share some common features but have distinct diagnostic and treatment approaches. We therefore undertook an updated systematic review and meta-analysis of observational studies reporting the incidence of overall complications, acute coronary syndromes, new or worsening heart failure, new or worsening arrhythmias and acute stroke, as well as short-term mortality outcomes. To set a framework for future research, we further included a holistic review of the interplay between the two conditions. From 1984 to 2019, thirty-nine studies were accrued, involving 92,188 patients, divided by setting (inpatients versus outpatients) and clinical severity (low risk versus high risk). Overall cardiac complications occurred in 13.9% (95% confidence interval (CI) 9.6–18.9), acute coronary syndromes in 4.5% (95% CI 2.9–6.5), heart failure in 9.2% (95% CI 6.7–12.2), arrhythmias in 7.2% (95% CI 5.6–9.0) and stroke in 0.71% (95% CI 0.1–3.9) of pooled inpatients. During this period, meta-regression analysis suggests that the incidence of overall and individual cardiac complications is decreasing. After adjusting for confounders, cardiovascular events taking place after community-acquired pneumonia independently increase the risk for short-term mortality (range of odds-ratio: 1.39–5.49). These findings highlight the need for effective, large trial based, preventive and therapeutic interventions in this important patient population.

## 1. Introduction

Community-acquired pneumonia (CAP) and cardiovascular (CV) disease are two major public health issues. CAP alone results in 1.7 million hospital admissions and almost 50,000 deaths every year in the USA [1,2], with a similar adjusted incidence observed in Europe [3,4]. In developed countries, CV disease is the leading cause of morbidity and mortality. Together, these conditions weigh heavily on national health systems and efforts to mitigate their global burden should be a priority for policy makers [5].

Although seemingly unrelated beforehand, the interplay between CAP and acute CV events has been elucidated in the last decades [6]. Myocardial infarction, heart failure, arrhythmias and stroke were witnessed to increase following a CAP episode [7]. The risk is maximal during the acute phase but persists in time after the infection has abated [8]. Moreover, a higher likelihood of a poorer outcome in a patient with CAP complicated by a CV event exists when compared to one with an uncomplicated course [7]. At the population level, the practical implications of such an association are immense. The widespread use of influenza and pneumococcal vaccination is an attractive option. When considering treatment possibilities, drugs with proven efficacy in the CV arena could theoretically be initiated to modulate outcomes, both acutely but also in the context of long-term CV prevention. Definite evidence-based guidance on how to perform these interventions at a patient level is, however, lacking.

In this article, we provide a broadened description of the current evidence associating CAP and acute CV disease. From bench to bedside, we begin with mechanistic links of the pathophysiologic repercussion of CAP on the cardiovascular system, proceed to an updated systematic review of and meta-analysis of observational clinical studies and end with the impact of potential interventions and future avenues of research. 

## 2. Materials and Methods

### 2.1. Search Strategy

We searched Medline from inception to 1 November 2019 for articles in English, French, Spanish, and Portuguese languages evaluating the incidence of subsequent CV events after a first CAP episode (community-acquired pneumonia” AND (“complications” OR “acute coronary syndromes” OR “myocardial infarction” OR “heart failure” OR “arrhythmia” OR “atrial fibrillation” OR “stroke”). To ensure the completeness of our review, we also retrieved previously published reviews on the subject and scanned their references for any additional missed publications in the primary search. 

### 2.2. Population and Outcomes

We restricted our search to adult non-immunocompromised patients with clinical and radiological evidence of CAP (as a means to improve specificity) for whom outcomes were explicitly reported in the methods section. Consecutive enrolment of patients and the quantification of CV events in the entire cohort were also prerequisites for inclusion. Studies focusing on nosocomial or health care–associated pneumonia, antibiotic efficacy trials (due to selection bias) and articles dealing primarily with pediatric patients or patients infected with the human immunodeficiency virus were excluded. 

If assessed, independent predictors for CV complications after CAP were collected and tabulated. To minimize the influence of patient heterogeneity in the estimation of effect sizes, we stratified patients according to treatment setting and clinical severity, when available. Patients initially treated in an ambulatory setting were defined as outpatients while patients admitted to a hospital on presentation were considered as inpatients. High risk patients were defined as having a pneumonia severity index (PSI) class IV or V or and/or requiring admission to the intensive care unit (ICU). Cardiovascular events were categorized as overall cardiac complications, acute coronary syndromes, new or worsening heart failure, and new or worsened arrhythmia and stroke. We collected data on all-cause mortality occurring either during hospital admission on during the first 30 to 90 days.

### 2.3. Study Selection, Data Extraction and Synthesis

**Figure 1:**
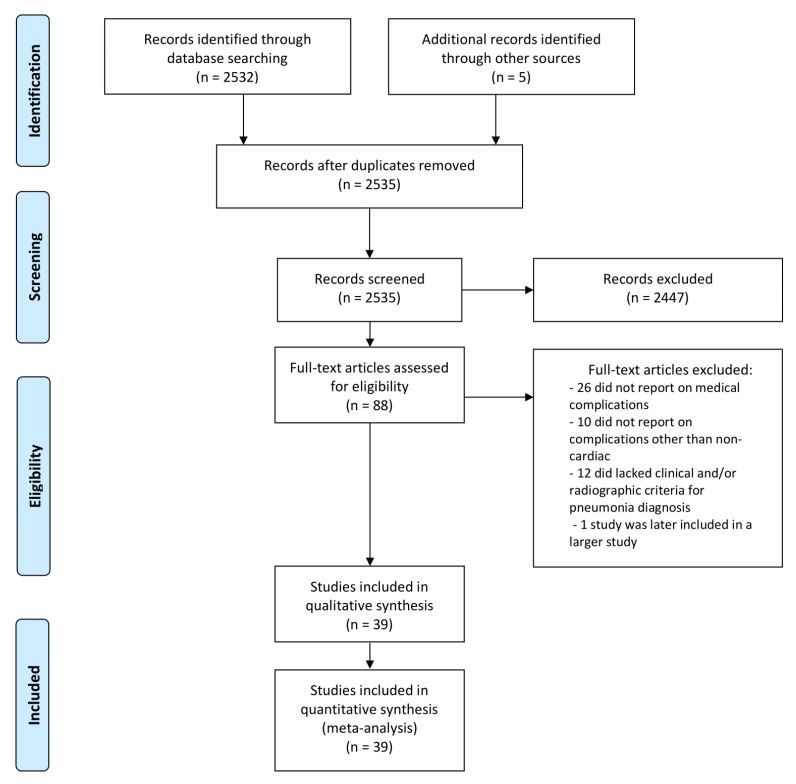
Study flowchart.

After the initial search, the first author extracted all abstracts, excluded irrelevant studies and if deemed adequate, proceeded to full-text reading and data tabulation. Study flowchart is depicted in Figure 1. The methodologic quality of available studies was graded according to Preferred Reporting Items for Systematic reviews and Meta-analyses (PRISMA) guidelines (Supplementary Table S1).

### 2.4. Statistical Analysis

In contrast to a traditional meta-analysis aiming to describe an effect size related to a therapeutic intervention, a meta-analysis of proportions has the goal of obtaining a more precise estimate of the overall proportion for a certain case or event [9]. In order to achieve this, raw proportional data need to be conformed to the normal distribution by employing one of several validated statistical approaches. We chose Freeman and Tukey’s double-arcsine transformation, a method which stabilizes study variance and reduces the probability of inaccurate weighting of each study when the inverse of the variance of the transformed proportion is used as a study weight [10]. To further minimize population differences between studies, patient cohorts were divided by event category, setting, and clinical severity before estimating pooled event rates using a random-effects model. 

We anticipated that the identification of more studies spanning the eight-year time frame after the last published systematic review would justify an analysis by year of publication. Therefore, a meta-regression using year of publication as a moderator variable was undertaken. To predict the effect of a hypothesized moderator, a weighted linear regression model was performed, in which the effect sizes (i.e., transformed proportions of CV events) are regressed onto the moderator, and a linear equation obtained. Finally, we assessed for publication bias by funnel plot inspection [11] and formally by Egger’s test [12]. In a funnel plot, each study is inscribed around the summary effect size (a vertical line) bounded by two converging slopes defining the 95% confidence interval around it. Imprecision is decrementally displayed on the y-axis and is zero at the triangle vertex. Absence of bias would result in a perfectly symmetrical plot. All analysis were performed using R (version 3.6.1, Vienna, Austria).

## 3. Results

### 3.1. Basic Science and Clinical Insights Associating CAP to CV Events

#### 3.1.1. Coronary Arteries and Myocardial Infarction

Theoretically, three different mechanisms prompted by pneumonia can contribute to a coronary event: (1) plaque rupture or fissuring leading to superimposed thrombus formation and cardiac troponin rise and/or fall, corresponding to type 1 myocardial infarction (MI); (2) type 2 MI, due to an imbalance between oxygen delivery and consumption in the myocardium, together with ischemic symptoms, especially if fixed stenoses are present, and (3) “myocardial injury”, when a troponin leak is detected without evidence of ischemia [13]. The most accepted and important pathway involves inflammation driven plaque instability [14]. By experimentally inducing sepsis in animal models, investigators have found post-mortem pathologic proof of atheromata growth and vascular inflammation. In mice fed with an atherogenic diet and subsequently developing provoked secondary fecal peritonitis, Kaynar et al. found increased abdominal aorta atheroma size, plaque neutrophil count and circulating proinflammatory cytokines versus a sham procedure, all persisting five months after the index intervention [15]. In humans, similarities are quite striking. In a post-mortem study, Mauriello et al. performed a detailed histologic analysis of coronary segments of sixteen patients who died after acute MI and compared them with controls who died of non-cardiac causes and had either stable angina or no CAD suspicion [16]. Significantly increased inflammatory infiltrates (macrophage and T-lymphocyte) were noticed not only in the culprit lesion but also in non-culprit vulnerable plaques and stable plaques in MI patients, suggesting underlying inflammation of the entire coronary tree even without sepsis. In another small sized study, fourteen patients who died from systemic infection (six of which with either upper or lower respiratory tract infection) were found to have more leucocytes in both plaque and adventitia than controls [17]. Inflammation that persists after the acute infectious episode could be causally linked increased mortality witnessed after hospital discharge following CAP [6].

Other proposed pathophysiologic pathways include hypercoagulability and hampered vascular tone. A significant proportion of pneumonia patients exhibited increased levels of coagulation markers in a study by Milbrandt et al, which persisted up to a week after hospital admission and were associated with increased mortality [18]. Although coronary perfusion is preserved or even increased in human septic shock [19], vasomotor disturbances resulting from changes in vasoactive mediators and capable of compromising myocardial oxygen delivery have been reported in animal [20] studies and could play a role in severe Gram positive infections.

#### 3.1.2. Myocardium and Heart Failure

Direct myocardial involvement by pathogens has been shown for viral and bacterial myocarditis caused mainly by influenza virus, adenovirus, respiratory syncytial virus and enterovirus but also bacteria including *Streptococcus pneumoniae*, *Chlamydophila pneumoniae*, *Mycoplasma pneumoniae*, *Staphylococcus aureus* and *Legionella* spp. [7]. Molecular studies have further suggested that up to a third of CAP cases in adults result from viral infection [7]. Kotaka et al. demonstrated that influenza virus inoculation in a murine model led to cytotoxic effect in cardiomyocytes and coronary thrombosis [21]. In the human heart, necropsy studies have similarly revealed edema, sarcomeric disarray and viral particle isolation from fatal influenza infection. Following invasive pneumococcal disease, Brown et al. disclosed for the first time how *Streptococcus pneumoniae* translocates into the myocardium of primates and patients who succumbed to CAP [22]. In their study, microlesions containing bacteria were described inside cardiomyocytes, which if treated with antibiotics led to local inflammation and scarring. The authors speculate that these foci may generate future myocardial dysfunction or cardiac arrhythmias.

#### 3.1.3. Cardiac Rhythm Disturbances

Atrial fibrillation is the most common sustained arrhythmia encountered in clinical practice [23]. Known transient risk factors include fever, hypoxia and hemodynamic disturbances, all of which can be triggered by infections including pneumonia [24]. An increased incidence in AF as well as other specified arrhythmias following CAP is a phenomenon well described in the literature. In a study conducted in critically ill patients, sepsis and acute respiratory failure were the most powerful predictors of AF onset [25]. Proposed mechanisms include direct toxic effect on the cardiac electric system, augmented loading imposed on cardiomyocytes, inflammation, sympathetic hyperactivity, ischemia and pharmacological agents, including antibiotics. AF occurrence is associated with worse short and long term outcomes in patients with infections [26,27], directly influencing patient management, namely the decision to initiate anticoagulation, and conditioning rhythm or rate control strategies.

### 3.2. Clinical Studies of Short Term Incident Acute CV Disease after CAP

We found 39 studies (Table 1, see also Supplementary Table S2 for detailed study description) reporting the incidence of CV events following CAP. Retrieved studies spanned a thirty-five-year period and included mostly North American patients. The mean weighted age of patients was 72 ± 11 years and most were male (79%).

#### 3.2.1. Overall Cardiac Complications

**Figure 2:**
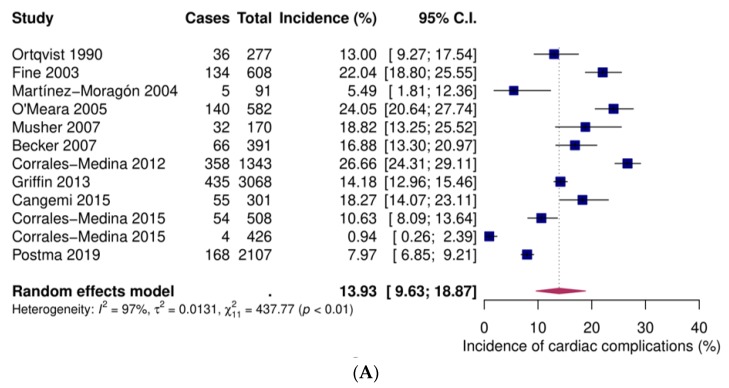

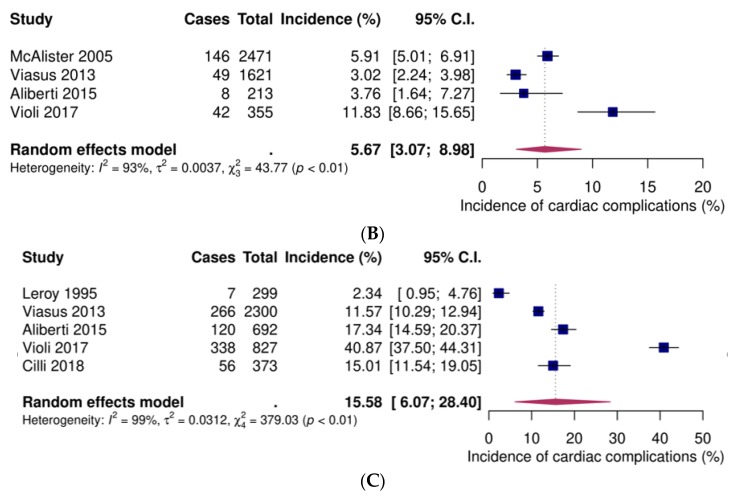
Forest plots of incident overall cardiac complications after community-acquired pneumonia. (**A**) Inpatients (**B**) Low-risk inpatients. (**C**) High-risk inpatients. CI: confidence interval.

Twelve studies provided data on inpatients [31,39,40,46,47,48,54,56,58,59,64], four on low-risk inpatients [45,55,57,61] and five on high-risk inpatients [35,55,57,61,63]. Pooled event rates were 13.9% (95% confidence interval (CI) 9.6–18.9), 5.7% (95% CI 3.1–9.0) and 15.6% (95% CI 6.1–28.4), respectively (Figure 2).

#### 3.2.2. Acute Coronary Syndromes

**Figure 3:**
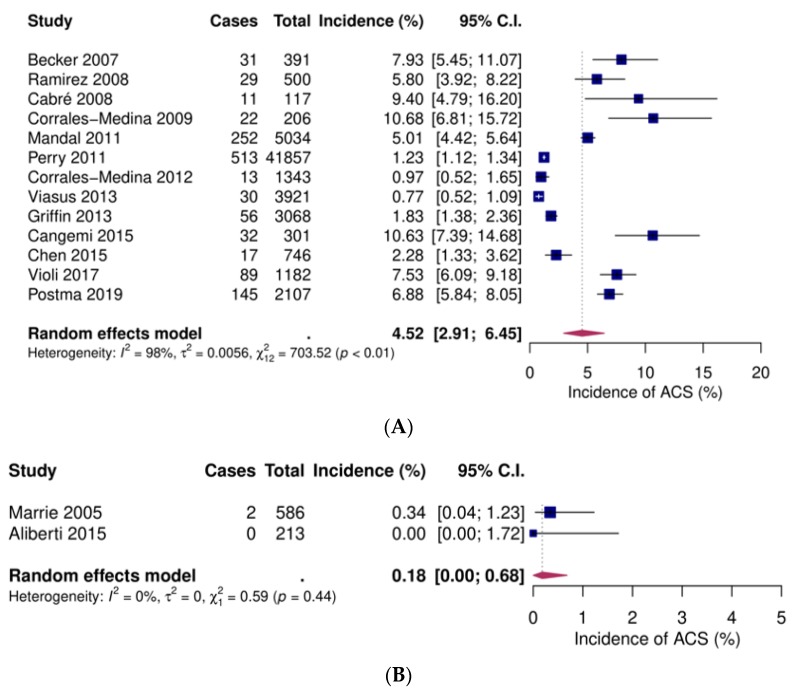

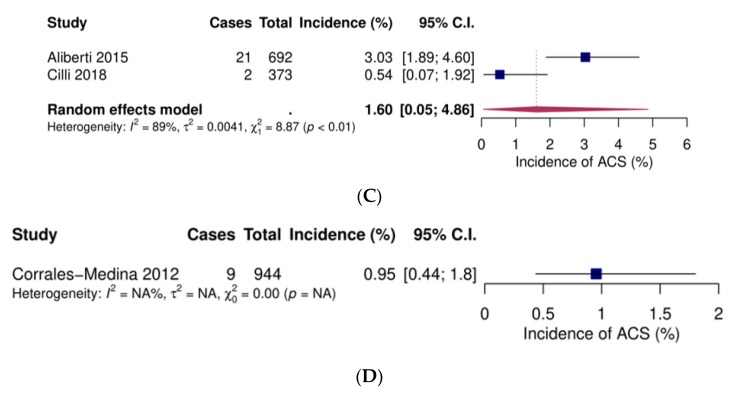
Forest plots of incident acute coronary syndromes (ACS) after community-acquired pneumonia. (**A**) Inpatients (**B**) Low-risk inpatients (**C**) High-risk inpatients (**D**) Outpatients. CI: confidence interval.

Thirteen studies reported data on inpatients [48,49,50,51,52,53,54,55,56,58,60,61,64], two on low-risk patients [44,57] and high-risk [57,63] patients and one on outpatients [54]. Pooled event rates were 4.5% (95% CI 2.9–6.5), 0.2 (95% CI 0–0.7), 1.6 (95% CI 0.1–4.9), and 0.9 (95% CI 0.5–1.8), respectively (Figure 3).

#### 3.2.3. Heart Failure

**Figure 4:**
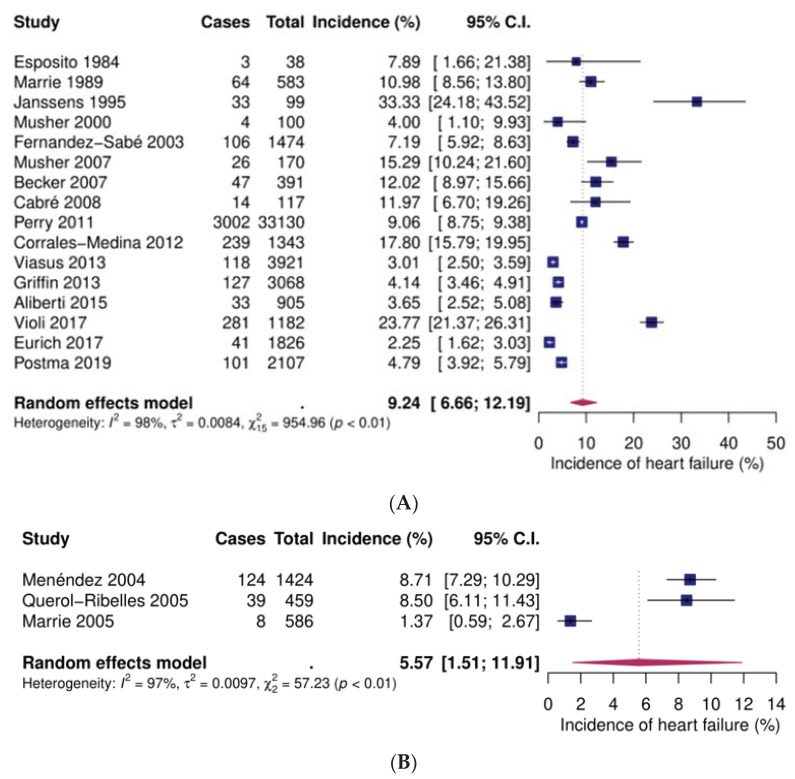

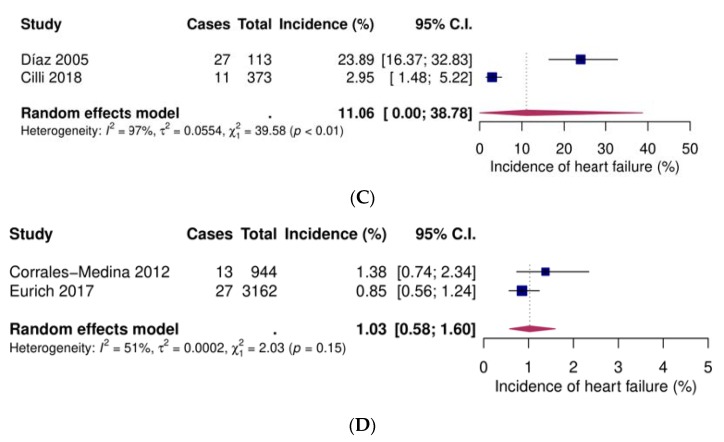
Forest plots of incident heart failure after community-acquired pneumonia. (**A**) Inpatients (**B**) Low-risk inpatients (**C**) High-risk patients (**D**) Outpatients. CI: confidence interval.

Sixteen studies included inpatients [29,30,36,37,38,47,48,50,53,54,55,56,57,61,62,64], three focused on low-risk patients [41,42,44], two focused on high-risk patients [43,63], and two on outpatients [54,62]. Pooled event rates were 9.2% (95% CI 6.7–12.2), 5.6% (95% CI 1.5–11.9), 11.1% (95% CI% 0–39), and 1.0% (95% CI 0.6–1.6), respectively (Figure 4).

#### 3.2.4. Arrhythmias

**Figure 5:**
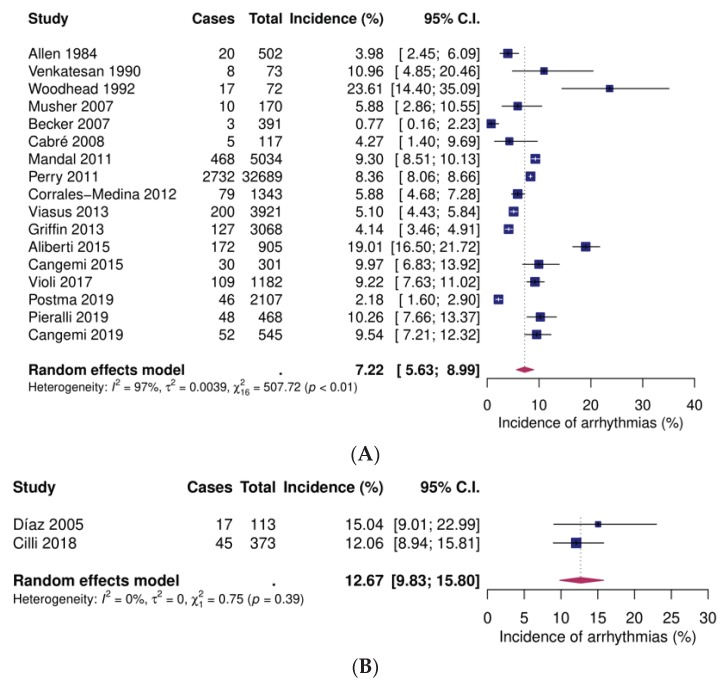

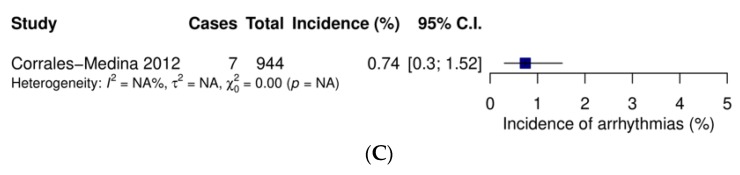
Forest plots of incident arrhythmias after community-acquired pneumonia. (**A**) Inpatients (**B**) High-risk inpatients (**C**) Outpatients. CI: confidence interval.

Incident arrhythmias were mentioned in twenty studies. Seventeen studies analysed inpatients [28,32,34,47,48,50,52,53,54,55,56,57,58,61,64,65,66], two focused on high-risk inpatients [43,63] and one on outpatients [54]. Pooled event rates were 7.2% (95% CI 5.6–9.0), 12.7% (95% CI 9.8–15.8), and 0.7% (95% CI 0.3–1.5), respectively (Figure 5).

#### 3.2.5. Stroke

**Figure 6:**
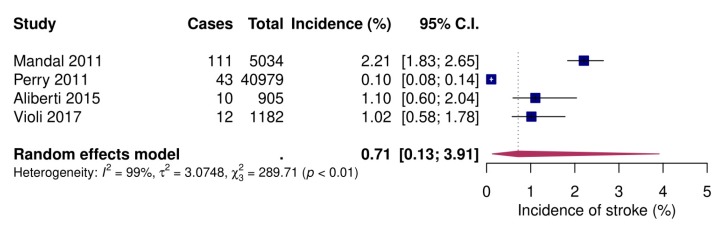
Forest plot of incident stroke of inpatients after community-acquired pneumonia. CI: confidence interval.

New-onset stroke following CAP was reported in four studies [52,57,61,63], all on inpatients. Pooled event rate was 1.7% (95% CI 1.0–2.6; Figure 6).

#### 3.2.6. Meta-Regression Analysis and Publication Bias Assessment

**Figure 7:**
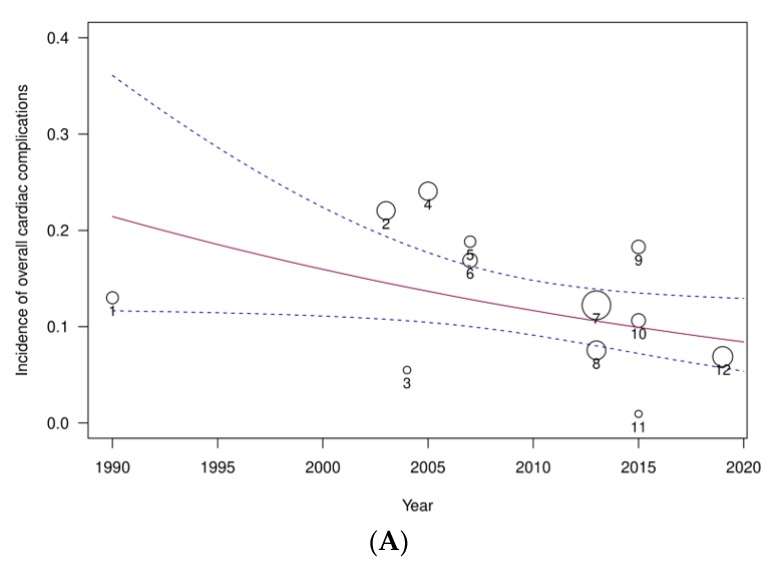

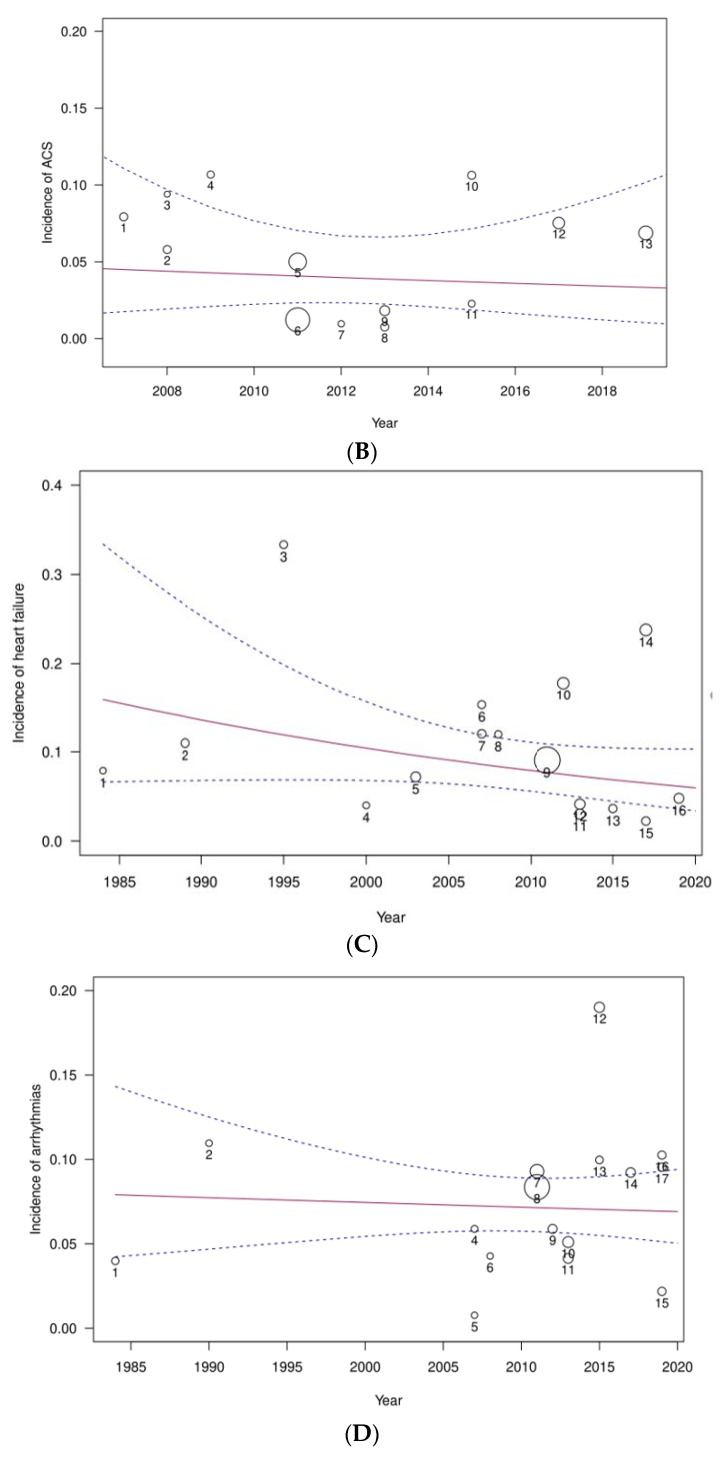
Meta-regression for (**A**) overall cardiac complications, (**B**) acute coronary syndromes ACS, (**C**) heart failure and (**D**) arrhythmias after community-acquired pneumonia, moderated by study year. Circle size is proportional to study sample size.

Meta-regression analysis (Figure 7) by study year revealed a decrease both in the overall proportion of cardiac complications and also individually when each complication was considered separately, more notably for incident heart failure. Funnel plot analysis and Egger’s test showed significant asymmetry for overall cardiac complications only (z = −3.7562, *p* = 0.0002; Supplementary File S1).

### 3.3. Risk Factors and Impact of Cardiac Complications on CAP Outcomes

Risk factors for the occurrence of CV events were reported in thirteen studies [51,52,53,54,55,56,57,58,61,63,64,65,66]. After adjustment for covariates, several clinical, microbiologic, laboratory, and imaging features were found to be independent predictors of an adverse CV event (Table 2).

Seven studies evaluated whether CV events were independently associated with mortality in CAP patients [52,54,55,57,58,61,63]. Multivariate analysis and propensity score matching were used to correct for confounders when assessing the impact of composite and individual CV events. Both overall and individual cardiac complications were associated with increased mortality after CAP (Table 3).

### 3.4. Long Tterm Outcomes after CAP 

Soon after the onset of infection, a plateau of maximum risk for CV events is reached and persists for the following 30 days [67]. Afterwards, it does not fall abruptly but gradually diminishes, conferring a long term increased risk that goes well beyond the acute phase [67]. By retrospectively analysing data from two large observational cohorts of patients without known CV disease, Corrales-Medina et al. have estimated almost a doubling of CV events during a ten-year period when compared to controls [59].

### 3.5. What Is the Role of Pharmacological Therapies?

Multiple investigators aimed to establish a role for CV and immunomodulating drug therapies in CAP patients. Below, we enumerate pharmacological classes and summarize the available evidence for their potential use in CAP.

#### 3.5.1. Antiplatelet Drugs

Different receptor pathways serve as potential targets for antiplatelet agents: (1) inhibitors of thromboxane A2 production (aspirin, triflusal), (2) antagonists of adenosine diphosphate (ADP)-activated P2Y_12_ receptors (ticlopidine, clopidogrel, prasugrel, ticagrelor, cangrelor), (3) antagonists of thrombin-activated proteinase activated receptor 1 (vorapaxar), and (4) GPIIb/IIIa inhibitors (abciximab, tirofiban, eptifibatide). Conflicting evidence regarding the role of aspirin in primary prevention led to the publication of two large scale randomized clinical trials who failed to demonstrate the benefit of aspirin in unselected patients without a previous CV event [68,69]. To make matters worse, a signal toward increased bleeding was detected, leaving no compelling evidence for the chronic blocking of platelet cyclooxygenase beyond secondary prevention. In CAP patients, two prospective propensity matched studies showed that aspirin may be associated with decreased CV events [70,71]. Recently, ticagrelor, a reversible P2Y_12_ platelet ADP receptor inhibitor, showed to decrease leukocyte adherent platelets and inflammatory markers in a small double-blinded trial in CAP [72]. Patients randomized to ticagrelor had faster amelioration of oxygenation deficit and a trend towards better progression in lung function test results. Although using an antiplatelet agent in the acute setting might seem appealing based on heightened inflammation during CAP, this hypothesis remains to be adequately tested in a powered randomized trial and cannot therefore be recommended.

#### 3.5.2. Statins and Other Lipid Lowering Agents

Impeding cholesterol synthesis is perhaps the most effective drug intervention to reduce the incidence of de novo and recurring CV events. Data from more that 170,000 patients have demonstrated a significant impact of statin therapy in all-cause mortality, CV mortality and CV events, with a reassuring safety profile, in both primary and secondary prevention settings [73]. In vitro and observational studies led to the belief that reducing inflammation through statins non-lipid lowering effects could translate into a similar benefit in sepsis, an hypothesis not confirmed in subsequent randomized trials [74]. Specifically, trials that tested statins in ventilator associated pneumonia [75] or acute respiratory distress syndrome [76] have not proved to be better than placebo, leaving no convincing role for their use in this setting. The need for a prolonged exposure to statins in order to obtain clinical benefit might be an explanation to justify failure to improve outcomes. Recently, two other lipid lowering drugs (evolocumab, alirocumab) acting through inhibition of PCSK9 (proproteine convertase subtilisin/kexin type 9, a protein that targets low-density lipoprotein receptors for degradation in the liver) achieved event reduction on top of statin therapy [77,78]. There are currently no published human studies exploring a potential effect of this new class of molecules on infection related outcomes. In an animal model, Berger et al. did not show a reduction of lipopolysaccharide induced mortality with the administration of anti-PCSK9 antibodies [79]. 

#### 3.5.3. Beta-Blockers, Angiotensin-Converting Enzyme Inhibitors (ACEi) and Angiotensin Receptor Blockers (ARB)

Given their established role in CV disease treatment, investigators have explored a possible benefit of other cardioprotective drugs in CAP patients. Wu et al. employed multilevel regression modeling to examine the association between CV drug classes and either mortality or CV events [80]. They found that while ACEi and ARBs were associated with decreased mortality, there was no significant association with decreased CV events. These results suggest that this decreased mortality is unlikely due to their potential cardioprotective effects. Higher quality studies are needed to confirm or refute this unexpected benefit.

#### 3.5.4. Corticosteroids

Corticosteroids have been tested in a wide spectrum of infectious diseases, yielding controversial results. In CAP, one large patient level meta-analysis [81] pointed to a mortality benefit (34% relative risk reduction), while in another meta-analysis [82] a very similar absolute reduction reached statistical significance with one of two random-effects models. In both studies, shorter length of stay (~1 day) and more hyperglycemic episodes were noted. In the analysis of Briel et al., steroids were associated with an increased incidence of CAP related readmissions for various reasons including CV events. Recently, Cangemi et al. found that using corticosteroids for CAP halved the incident of intra-hospital MI, albeit not reducing either all-cause or CV mortality [83]. 

#### 3.5.5. Novel Drugs

Canakinumab, a fully human monoclonal antibody targeting 1L-ß interleukin demonstrated a reduction in recurrent CV events in a randomized clinical trial against placebo [84]. Conversely, the drug was associated with a small but significant increase of fatal infection or sepsis. Pneumonia rates were similar in both treatment arms. 

### 3.6. Vaccination

Observational studies and secondary post-hoc analysis of large trials in CV disease have hinted towards a prognostic impact of influenza and pneumococcal vaccination, particularly in coronary artery disease related outcomes [85]. In a meta-analysis of five randomized trials, influenza vaccination was associated with a 36% relative risk reduction in the incidence of major adverse cardiovascular events during a 12-month period, with no influence in mortality [86]. Patients who had a recent ACS seemed to derive the largest benefit (55% relative risk reduction). Inferior quality evidence exists for pneumococcal vaccination. A meta-analysis of observational studies by Ren et al. revealed a 23% reduction in ACS in patients aged 65 or above [87]. The benefit was lost when all patients were considered and was not extensible to other vascular territories. In the heart failure population, a signal towards a reduction in a composite endpoint of CV mortality and HF hospitalization in propensity matched influenza-vaccinated patients from a large trial was identified [88]. There are presently no studies specifically addressing the effect of pneumococcal immunisation in HF patients.

### 3.7. Effects of Antibiotics on the Cardiovascular System

While decisive to improve outcomes in patients with CAP, antibiotics are not free from toxicity, which may include the CV system. Although possessing useful anti-inflammatory properties, macrolides have been ascribed an increased risk in arrhythmic events and sudden cardiac death (SCD) through QT interval prolongation and polymorphic ventricular tachycardia facilitation. For azithromycin, the absolute risk was estimated to be an additional 47 deaths per one million five-day courses of therapy (all indications considered) when compared to amoxicillin [89]. In a smaller propensity score matching study, Schembri et al. also found an independent association between clarithromycin use for CAP and CV events during a one-year follow-up (HR 1.68, 95% CI 1.18–2.38), which was not extensible to increased mortality [90].

## 4. Discussion 

By analysing epidemiologic data from more than a quarter billion individuals, Collins found an excess all-cause mortality accompanying a pairwise increase in influenza and pneumonia related fatalities during successive early 20^th^ century infection outbreaks [91]. After this seminal report, a burgeoning number of observational studies and reviews has strengthened this association [92,93,94,95,96]. Our updated systematic review and meta-analysis, encompassing 92,188 patients, the overwhelming majority (95%) treated as inpatients, further emphasizes the burden of incident CV disease following CAP. After pooling individual study rates, we found an incidence of overall cardiac complications of 13.9%. The most frequent cardiac complication in inpatients who were not stratified by severity was de novo or worsening heart failure in 9.2% of patients, 7.2% new-onset or worsening arrhythmias, and ACS in 4.5%. Stroke was the least common CV event in CAP patients. 

The present review accrues data from fourteen more additional studies (~75,000 patients) to the last review by Corrales-Medina et al. [97], allowing us to perform a supplementary meta-regression moderated by study year. Globally and individually, the incidence of acute CV events following CAP seems to be decreasing, albeit slightly. These findings seem to be in line with reported rates of CV events in the non-CAP population, with the exception of atrial fibrillation. Yearly trends in acute myocardial infarction show that hospital admission is decreasing in both the US and in Europe [5,98]. This may reflect the improved utilization of protective CV medication in the context of better primary care based prevention including pneumococcal and influenza immunization, along with improved secondary prevention after ACS. For heart failure, statistics indicate that the incidence is diminishing, at the expense of increased prevalence, which can be attributed to demographic shifts, improved quality of care, medical therapy and handling of comorbidities [99]. Finally, regarding atrial fibrillation, data published by Schnabel et al. suggest that both its incidence and prevalence are increasing [100]. In our study, the influence of considering all arrhythmias might have contributed to this apparent discrepancy.

After incorporating more recent studies, the list of independent predictors for cardiac complications is now longer and more complex. The range of predisposing factors includes host (CV and non-CV comorbidities) and pathogen-related features. Whilst the baseline comorbidities may share the same approach and in a way merely denote a sicker patient, different microbes may pose an opportunity for influencing patient outcomes by improving time to diagnosis and starting pathogen specific interventions. For pneumococcal pneumonia, bacterial exotoxin pneumolysin has been identified as key effector of cell and organ damage in murine models [101,102] and could theoretically become a target for macrolides (or macrolide-like antibiotics), statins or cholesterol rich liposomes [103]. If influenza is being considered, prompt detection may allow the early initiation of neuraminidase inhibitors. Not surprisingly, if a CV event supervenes after CAP, patients will likely fare worse. Our aggregated data from eight studies suggests an up to five-fold increase in mortality, mostly for compiled cardiac complications but also for individual events. Despite its observational nature, this signal is consistent between studies and possesses biological credibility, anticipating the likely presence of a true effect. 

Notwithstanding the strengths provided by the increased number of studies included and a clearer view on the temporal trends in CV events after CAP in our updated review and meta-analysis, some limitations have to be acknowledged. Population heterogeneity, different causal pathogens, asymmetries in study design and imprecise event definitions may be responsible for the observed between study variance and wide confidence intervals around the measured effect, reducing the accuracy estimates of the real effect. Funnel plot analysis is in agreement with such wide dispersion of data. For heart failure, the interpretation of results is confounded by the fact that both diseases are simultaneously risk factors and consequences of each other. Finally, we chose not to perform a meta-regression of stroke due to the paucity of data on acute cerebrovascular events after CAP.

## 5. Future Directions

Despite the apparent reduction in the event rate, the absolute burden of CV disease means there is clearly room for improvement. Hence, from a health system perspective, CV morbidity and mortality after CAP highlight the pressing need for (1) acquiring a better understanding of the pathophysiologic mediators that lead to acute CV events, (2) conducting well-designed randomized clinical trials to assess the effect of targeted drug interventions (e.g., statins) on acute CV outcomes in CAP patients, and (3) implementing a risk stratification model to ameliorate the long-term prognosis of these patients. Finally, and despite common features shared by myocardial infarction, heart failure, arrhythmias, and stroke, gaining deeper insight into the interaction between each event and CAP could prove useful in tailoring preventative and therapeutic strategies at a patient level.

## Figures and Tables

**Table 1:** Characteristics of included studies.

-	Year	Country	Study Type	Setting	*n*	CV Events ^a^ (%)	ACS ^b^ (%)	Heart Failure (%)	Stroke ^c^ (%)	Arrhythmias ^d^ (%)
Allen et al. [28]	1984	Zambia	Retrospective single center	Inpatients	502	-	-	-	-	0.40
Esposito et al. [29]	1984	USA	Prospective single center	Inpatients	38	-	-	7.9	-	-
Marrie et al. [30]	1989	Canada	Prospective single center	Inpatients	583	-	-	11	-	-
Ortqvist et al. [31]	1990	Sweden	Prospective, single center	Inpatients	277	13	-	-	-	-
Venkatesan et al. [32]	1990	UK	Prospective single center	Inpatients	73	-	-	-	-	11
Fine et al. [33]	1990	USA	Prospective single center	Low-risk ^e^ inpatients Outpatients	170	-	0.6	-	-	0.6
Woodhead et al. [34]	1992	UK	Retrospective multicenter	High-risk ^f^ inpatients	72	-	-	-	-	23
Leroy et al. [35]	1995	France	Retrospective single center	High-risk ^f^ inpatients	299	2.3	-	-	-	-
Janssens et al. [36]	1995	Switzerland	Prospective, single center	Inpatients	99	-	-	33	-	-
Musher et al. [37]	2000	USA	Prospective single center	Inpatients	100	-	-	4.0	-	-
Férnandez-Sabé et al. [38]	2003	Spain	Prospective single center	Inpatients	1474	-	-	7.2	-	-
Fine et al. [39]	2003	USA	Prospective multicenter	Inpatients	608	22	-	-	-	-
Martínez-Moragón et al. [40]	2004	Spain	Prospective single-center	Inpatients	91	5.0	-	-	-	-
Menéndez et al. [41]	2004	Spain	Prospective multicenter	Low-risk ^e^ inpatients	1424	-	-	8.7	-	-
Querol-Ribelles et al. [42]	2005	Spain	Prospective single-center	Low-risk ^e^ inpatients	459	-	-	8.6	-	-
Díaz et al. [43]	2005	Chile	Prospective Single-center	High-risk ^f^ inpatients	113	-	-	24	-	15
Marrie et al. [44]	2005	Canada	Prospective single center	Low-risk ^e^ inpatients	586	-	0.3	1.4	-	-
McAlister et al. [45]	2005	Canada	Prospective multicenter	Low-risk ^e^ inpatients	2471	5.9	-	-	-	-
O’Meara et al. [46]	2005	USA	Prospective multicenter	Inpatients	582	24	-	-	-	-
Musher et al. [47]	2007	USA	Retrospective single-center	Inpatients ^g^	170	19	7	15	-	6
Becker et al. [48]	2007	Canada	Retrospective multicenter	Inpatients	391	17	8	12	-	3
Ramirez et al. [49]	2008	Spain	Retrospective single-center	Inpatients	500	-	5.8	-	-	-
Cabré et al. [50]	2008	Spain	Prospective single-center	Inpatients	117	-	0.9	12	-	4.4
Corrales-Medina et al. [51]	2009	USA	Retrospective single-center	Inpatients	206	-	11	-	-	-
Mandal et al. [52]	2011	Scotland	Retrospective multicenter	Inpatients	5034	-	5.0	-	2.2	9.3
Perry et al. [53]	2011	USA	Retrospective multicenter	Inpatients	50119	-	2.3	9.1	0.1	8.4
Corrales-Medina et al. [54]	2012	USA and Canada	Prospective multicenter	Inpatients Outpatients	1343 944	27 2.1	3.6 0	67 65	-	22 35
Viasus et al. [55]	2013	Spain	Prospective single center	Low-risk inpatients High-risk inpatients ^f^	1621^h^ 2300	3.0 11.6	0.76	3.0	-	5.1
Griffin et al. [56]	2013	13 countries	Retrospective multicenter	Inpatients	3068	14	1.3	2.1	-	3.6
Aliberti et al. [57]	2015	Italy, Switzerland	Retrospective multicenter	Inpatients	905	-	2.3	3.7	1.1	19
Cangemi et al. [58]	2015	Italy	Prospective, single center	Inpatients	301	18	11	-	-	10
Corrales-Medina et al. [59]	2015	USA	Retrospective multicenter	Inpatients	508	11	-	-	-	-
Corrales-Medina et al. [59]	2015	USA	Retrospective multicenter	Inpatients	426	0.90	-	-	-	-
Chen et al. [60]	2015	Taiwan	Single-center retrospective	Inpatients	746	-	2.3	-	-	-
Violi et al. [61]	2017	Italy, Canada	Prospective multicenter	Low-risk inpatients High-risk inpatients ^f^	355 ^h^ 827	12 41	8.4	24	0.1	9.2
Eurich et al. [62]	2017	Canada	Prospective multicenter	Inpatients Outpatients	4988	-	-	12	-	-
Cilli et al. [63]	2018	Turkey	Retrospective multicenter	High-risk ^f^ inpatients	373	15	0.54	2.9	-	12
Postma et al. [64]	2019	Netherlands	Retrospective multicenter	Inpatients	2107	7.9	0.7	4.8	-	2.5
Pieralli et al. [65]	2019	Italy	Retrospective single-center	Inpatients	468	-	-	-	-	10.3
Cangemi et al. [66]	2019	Italy	Prospective single center	Inpatients	545	-	-	-	-	9.5

^a^ Cardiovascular (CV) events: congestive heart failure, atrial fibrillation, severe angina or myocardial infarction or stroke [31]; acute coronary or ventricular insufficiency [35]; cardiovascular complications likely to necessitate continued hospitalization [39]; cardiac complications without further specification [40]; acute coronary syndrome and/or heart failure [45]; myocardial infarction, angina pectoris, revascularization by angioplasty/coronary artery bypass graft (CABG) or death secondary to coronary heart disease, cerebrovascular accident, congestive heart insufficiency or claudication [46]; myocardial infarction, atrial fibrillation or ventricular tachycardia or incident heart failure [47]; myocardial infarction, atrial fibrillation, congestive heart failure or stroke [48]; new or worsening heart failure, new or worsening arrhythmias or myocardial infarction [54]; new-onset or worsening cardiac arrhythmias, new-onset or worsening congestive heart failure or myocardial infarction [55]; acute pulmonary edema, new onset cardiac arrhythmia, exacerbation of a preexisting arrhythmia, or myocardial infarction [56]; acute myocardial infarction, acute cardiogenic pulmonary edema, new arrhythmia, acute worsening of a long-term arrhythmia, cerebrovascular accident or pulmonary embolism [57];cardiovascular death, non-fatal myocardial infarction or stroke [58]; non-ST elevation myocardial infarction or ST elevation myocardial infarction, stroke, new episode of atrial fibrillation or deep venous thrombosis and/or pulmonary embolism, new or worsening HF or cardiovascular death [61] new onset or worsening arrhythmia, new onset or worsening heart failure or myocardial infarction [63]: new or worsening arrhythmia, heart failure or myocardial ischemia [64]. ^b^ Acute coronary syndromes (ACS): myocardial infarction [33,37,39,47,48,49,53,54,55,56,57,58,60,61,63,64]; unstable angina [44]; acute coronary syndrome [50,51]; acute coronary syndrome or ST segment elevation myocardial infarction [52]. ^c^ Stroke: new-onset neurological deficit [53]; unspecified stroke [52,61]; cerebrovascular accident [57]. ^d^ Arrhythmias: incident atrial fibrillation [28,32,33,48,49,50,52,53,54,55,56,57,58,61,64,65,66]; cardiac dysrhythmias/arrhythmias [34,43]; atrial flutter or fibrillation, and ventricular tachycardia, but excluding terminal arrhythmias [47]. ^e^ Inpatients without severe vital signs or metabolic abnormalities, altered mental status, suppurative complications or coexisting medical conditions requiring hospitalization [33]; inpatients who survived the first 48 h of hospitalization [41], inpatients not initially admitted to the intensive care unit [42,45]; inpatients with pneumonia severity index (PSI) risk classes I–II [44]. ^f^ Inpatients admitted to the intensive care unit (ICU). ^g^ For ACS, patients from Musher et al. (2007) [47] were included in Corrales-Medina et al. (2009) [51]. ^h^ Data available for low-risk or high-risk patients if overall cardiac events are considered.

**Table 2:** Included studies presenting independent predictors of cardiovascular (CV) events after community-acquired pneumonia (CAP).

Author	Year	*n* with Event	Outcome	Independent Predictors
Corrales-Medina et al. [51]	2009	206	ACS	Age Congestive heart failure
Mandal et al. [52]	2011	252	ACS	Age ≥ 65 Previous MI COPD Chronic kidney disease
		468	Arrhythmias	Age Previous MI Diabetes
		111	Stroke	Prior stroke COPD
Perry et al. [53]	2011	2002	CHF	Age Admission to ICU Previous MI COPD Diabetes Chronic kidney disease Cancer
		2732	Arrhythmias	Age Admission to ICU
Corrales-Medina et al. [54]	2012	378	Overall cardiac complications	Age Nursing home Hypertension Previous CAD Previous arrhythmias Previous CHF RR ≥ 30/min pH < 7.35 BUN ≥ 30 mg/dL Sodium < 130 mmol/L Hematocrit < 30% Pleural effusion Inpatient
Viasus et al. [55]	2013	315	Overall cardiac complications	Age ≥ 65 Chronic heart disease Septic shock Tachycardia Albumin < 3 g/dL Multilobar pneumonia Streptococcal pneumonia
Griffin et al. [56]	2013	376	Overall cardiac complications	Hyperlipidemia Statin therapy ^1^ *Staphylococcus aureus* *Klebsiella pneumoniae* PSI
Aliberti et al. [57]	2015	21	ACS	Female sex Severe sepsis Liver disease
Cangemi et al. [58]	2015	55	Overall cardiac complications	Age Hypertension Diabetes Baseline troponin
Violi et al. [61]	2017	308	Overall cardiac complications	Age CHF PSI
Cilli et al. [63]	2018	56	Overall cardiac complications	Age Hypoalbuminemia Diuretic Vasopressor Haloperidol
Postma et al. [64]	2019	2107	Overall cardiac complications	Erythromycin use
Pieralli et al. [65]	2019	468	Atrial fibrillation	CURB-65 > 2 CHA_2_DS_2_-VASc > 3
Cangemi et al. [66]	2019	545	Atrial fibrillation	Prior paroxysmal AF, Enlarged LAAi Left ventricular hypertrophy

^1^ Protective effect. ACS: acute coronary syndrome; AF: atrial fibrillation; BUN: blood urea nitrogen; CAD: coronary artery disease; CHA_2_DS_2_-VASc: congestive heart failure, hypertension, age-doubled, diabetes, stroke-doubled, vascular disease, age, sex-category; CHF: congestive heart failure; COPD: chronic obstructive pulmonary disease; CURB-65: confusion, urea, respiratory rate, blood urea nitrogen, age > 65; ICU: intensive care unit; LAAi (left atrial area index); MI: myocardial infarction; PSI: pneumonia severity index; RR: respiratory rate.

**Table 3:** Association of CV events and mortality after CAP.

Study	Year	Event	Mortality	Measure of Risk
**Mandal et al.** [52]	2001	Stroke	90-day	OR 1.79 (1.51–2.12), *p* < 0.0001
		MI		OR 2.93 (1.60–2.33), *p* < 0.0001
		AF		OR 1.39 (1.65–2.19), *p* < 0.0001
**Corrales-Medina et al.** [54]	2012	Overall cardiac complications	30-day	OR 1.6 (1.04–2.5), *p* < 0.001
**Viasus et al.** [55]	2013	Overall cardiac complications	30-day	OR 2.18 (1.38–3.42)
**Aliberti et al.** [57]	2015	ACS	In-hospital	OR 3.57 (1.32–9.69), *p* = 0.02
		Other events		OR 2.63 (1.43–4.84), *p* = NS
**Cangemi et al.** [58]	2015	Overall cardiac complications	6-60 months	OR 1.759 (1.099–2.816), *p* = 0.019
**Violi et al.** [61]	2017	Overall cardiac complications	30-day	HR 5.49, *p* < 0.001
**Cilli et al.** [63]	2018	Overall cardiac complications	In-hospital	OR 2.18 (1.03–4.61), *p* = 0.04
			90-day	NS

ACS: acute coronary syndrome; AF: atrial fibrillation; MI: myocardial infarction; NS: not significant; OR: odds-ratio; HR: hazard-ratio.

## Supplementary Materials

The following are available online at https://www.mdpi.com/2077-0383/9/2/414/s1. Table S1: PRISMA checklist; Table S2: Detailed characteristics of included studies; File S1: Funnel plot and Egger’s test for included studies of (a) overall cardiac complications, (b) ACS, (c) heart failure, (d) arrhythmias and (e) stroke after CAP.
