# Supplementary material for: Cardiovascular Events after Community-Acquired Pneumonia: A Global Perspective with Systematic Review and Meta-Analysis of Observational Studies
Source: J Clin Med. 2020 Feb 3;9(2):414. doi: 10.3390/jcm9020414 (PMC7073946; doi:10.3390/jcm9020414)
Supplement: Supplementary file 1 [file jcm-09-00414-s001.zip › Supplementary table S2_rev.docx]

| Reference | Year | Country | Study type | Setting | n | Age  (mean) | Male gender  (%) | Previous cardiovascular disease | | | | Mortality  (%) | CV  Events^a^  (%) | ACS^b^  (%) | Stroke^c^  (%) | Heart failure (%) | Arrhythmias^d^  (%) |
| --- | --- | --- | --- | --- | --- | --- | --- | --- | --- | --- | --- | --- | --- | --- | --- | --- | --- |
|  |  |  |  |  |  |  |  | Coronary artery disease  (%) | CBV disease  (%) | Heart failure  (%) | Arrhythmias  (%) |  |  |  |  |  |  |
| Allen *et al* [28] | 1984 | Zambia | Retrospective single center | Inpatients | 502 | 39 | 71 | - | - | - | - | 5.6  (in-hospital) | - | - | - | - | 0.4 |
| Esposito *et al* [29] | 1984 | USA | Prospective single center | Inpatients | 38 | 61 | 40 | 29 | - | - | - | 13  (in-hospital) | - | - | - | 7.9 | - |
| Marrie *et al* [30] | 1989 | Canada | Prospective single center | Inpatients | 583 | 60 | 65 | 25 | - | - | - | 21  (in-hospital) | - | - | - | 11 | - |
| Ortqvist *et al* [31] | 1990 | Sweden | Prospective single center | Inpatients | 277 | 62 | 43 | - | - | - | - | 4  (in-hospital) | 13 | - | - | - | - |
| Venkatesan *et al* [32] | 1990 | UK | Prospective single center | Inpatients | 73 | 79 | 52 | - | - | - | - | 22  (14-day) | - | - | - | - | 11 |
| Fine *et al* [33] | 1990 | USA | Prospective  single center | Low-risk inpatients^e^ Outpatients | 76  94 | 52 | 56 | 15 | - | 6 | - | 3.5  (6-week) | - | 0.6 | - | - | 0.6 |
| Woodhead *et al* [34] | 1992 | UK | Retrospective multicenter | High-risk inpatients^f^ | 72 | 54 | 55 | - | - | - | - | 48  (in-hospital) | - | - | - | - | 23 |
| Leroy *et al* [35] | 1995 | France | Retrospective  single center | High-risk inpatients^f^ | 299 | 64 | 63 | - | - | - | - | 28  (ICU) | 2.3 | - | - | - | - |
| Janssens *et al* 366] | 1995 | Switzerland | Prospective single center | Inpatients | 99 | 85 | 36 | - | - | - | - | 14  (30-day) | - | - | - | 33 | - |
| Musher *et al*  [37] | 2000 | USA | Prospective single center | Inpatients | 100 | 62 | 98 | - | - | 22 | - | 17  (30-day) | - | - | - | 4.0 | - |
| Férnandez-Sabé *et al* [38] | 2003 | Spain | Prospective single center | Inpatients | 1474 | 65 | 70 | - | 1.9 | - | - | 8.2  (30-day) | - | - | - | 7.2 | - |
| Fine *et al* [39] | 2003 | USA | Prospective,  multicenter | Inpatients | 608 | 69 | 46 | 38 | 17 | 23 | - | 8.5  (30-day) | 22 | - | - | - | - |
| Martínez-Moragón *et al* [40] | 2004 | Spain | Prospective single-center | Inpatients | 91 | 75 | 44 | - | - | 15 | - | 11  (30-day) | 5.0 | - | - | - | - |
| Menéndez *et al* [41] | 2004 | Spain | Prospective multicenter | Low-risk inpatients^e^ | 1424 | 68 | 67 | - | - | - | - | 5.6  (30-day) | - | - | - | 8.7 | - |
| Querol-Ribelles *et al* [42] | 2005 | Spain | Prospective single-center | Low-risk inpatients^e^ | 459 | 71 | 71 | 13 | - | 17 | - | 8.7  (30-day) | - | - | - | 8.6 | - |
| Díaz *et al* [43] | 2005 | Chile | Prospective  Single-center | High-risk inpatients^f^ | 113 | 73 | 58 | - | - | - | - | 17  (30-day) | - | - | - | 24 | 15 |
| Marrie *et al* [44] | 2005 | Canada | Prospective, single center | Low-risk inpatients^e^ | 586 | 47 | 41 | - | - | - | - | 0.9  (30-day) | - | 0.3 | - | 1.4 | - |
| McAlister *et al* [45] | 2005 | Canada | Prospective  multicenter | Low-risk inpatients^e^ | 2471 | 75 | 52 | - | 12 | 18 | - | 9.0  (30-day) | 5.9 | - | - | - | - |
| O’Meara *et al* [46] | 2005 | USA | Prospective  multicenter | Inpatients | 582 | 75 | 49 | 30 | 10 | 9 | - | 12  (in-hospital) | 24 | - | - | - | - |
| Musher *et al* [47] | 2007 | USA | Retrospective single-center | Inpatients | 170 | NR | >95 | - | - | - | - | 12  (in-hospital) | 19 | 7 | - | 15 | 6 |
| Becker *et al* [48] | 2007 | Canada | Retrospective multicenter | Inpatients | 391 | 77 | 50 | 23 | - | 18 | - | 11  (in-hospital) | 17 | 8 | - | 12 | 3 |
| Ramirez *et al* [49] | 2008 | Spain | Retrospective single-center | Inpatients | 500 | 70 | 98 | 43 | - | 26 | - | 11  (30-day) | - | 5.8 | - | - | - |
| Cabré *et al* [50] | 2008 | Spain | Prospective, single-center | Inpatients | 117 | 85 | 59 | 15 | - | 26 | - | 13  (30-day) | 17 | 0.9 | - | 12 | 4.4 |
| Corrales-Medina *et al* [51] | 2009 | USA | Retrospective single-center | Inpatients | 206 | 68 | >95 | 33 | - | 19 | - | 12  (30-day) | - | 11 | - | - | - |
| Mandal *et al* [52] | 2011 | Scotland | Retrospective multicenter | Inpatients | 5034 | 73 | 48 | 8 | 19 | 11 | - | 14  (90-day) | - | 5.0 | 2.2 | - | 9.3 |
| Perry *et al* [53] | 2011 | USA | Retrospective multicenter | Inpatients | 50119 | 78 | 98 | 17 | 18 | 34 | 35 | - | - | 2-3 | 0.1 | 9.1 | 8.4 |
| Corrales-Medina *et al* [54] | 2012 | USA,  Canada | Prospective multicenter | Inpatients  Outpatients | 1343  944 | 64  45 | 52  47 | 26  6 | 14  2 | 17  3 | 17  2 | 4.8;  (30-day) | 27  2.1 | 3.6  0 | - | 67  65 | 22  35 |
| Viasus *et al* [55] | 2013 | Spain | Prospective single center | Low-risk patients  High-risk patients | 1621^h^  2300 | 69 | 69 | - | 7.6 | - | - | 7.5  (30-day) | 3.0  11.6 | 0.76 | - | 3.0 | 5.1 |
| Griffin *et al* [56] | 2013 | 13 countries | Retrospective multicenter | Inpatients | 3068 | 66 | 58 | - | - | - | 7.9 | 13  (28-day) | 12 | 1.3 | - | 2.1 | 3.6 |
| Aliberti *et al* [57] | 2015 | Italy, Switzerland | Retrospective multicenter | Inpatients | 905 | 77 | 58 | 18 | 22 | 20 | 16 | 9.0  (in-hospital) | - | 2.3 | 1.1 | 3.7 | 19 |
| Cangemi *et al* [58] | 2015 | Italy | Prospective single center | Inpatients | 301 | 72 | 62 | - | 12 | - | 26 | 30  (6-60 months) | 18 | 11 | - | - | 10 |
| Corrales-Medina *et al* [59] | 2015 | USA | Retrospective multicenter, | Inpatients | 508 | 74 | 42 | - | - | - | - | - | 11 | - | - | - | - |
| Corrales-Medina *et al* [59] | 2015 | USA | Retrospective multicenter | Inpatients | 426 | 56 | 46 | - | - | - |  | - | 0.90 | - | - | - | - |
| Chen *et al* [60] | 2015 | Taiwan | Single-center retrospective | Inpatients | 746 | 78 | 64 | - | 33 | 16 | - | 4.2  (in-hospital) | - | 2.3 | - | - | - |
| Violi *et al* [61] | 2017 | Italy  Canada | Prospective  multicenter | Low-risk patients  High-risk patients | 355^h^  827 | 73 | 59 | 38 | 12 | 29 | 31 | 8.7  (30-day) | 12  41 | 8.4 | 0.1 | 24 | 9.2 |
| Eurich *et al* [62] | 2017 | Canada | Prospective  multicenter | Inpatients  Outpatients | 1826  3162 | 55 | 53 | - | - | - | 6.2 | 38  (9.6 years) | - | - | - | 12 | - |
| Cilli *et al* [63] | 2018 | Turkey | Retrospective multicenter | High-risk inpatients^f^ | 373 | 68 | 61 | - | - | 8.8 | 11 | 14  (in-hospital) | 15 | 0.54 | - | 2.9 | 12 |
| Postma *et al* [64] | 2019 | Netherlands | Retrospective multicenter | Inpatients | 2107 | 69 | 58 | 20 | 22 | 9.4 | 14 | - | 6.9 | 0.7 | - | 4.8 | 2.5 |
| Pieralli *et al* [65] | 2019 | Italy | Retrospective single-center | Inpatients | 468 | 76 | 48 | - | - | - | - | 12  (in-hospital) | - | - | - | - | 10.3 |
| Cangemi *et al* [66] | 2019 | Italy | Prospective single center | Inpatients | 545 | 70 | 63 | - | - | - | - | - | - | - | - | - | 9.5 |

^a^ **^Cardiovascular events^**^: congestive heart failure, atrial fibrillation, severe angina or myocardial infarction or stroke [31]; acute coronary or ventricular insufficiency [35]; cardiovascular complications likely to necessitate continued hospitalization [39]; cardiac complications without further specification [40]; acute coronary syndrome and/or heart failure [45]; myocardial infarction, angina pectoris, revascularization by angioplasty/coronary artery bypass graft (CABG) or death secondary to coronary heart disease, cerebrovascular accident, congestive heart insufficiency or claudication [46]; myocardial infarction, atrial fibrillation or ventricular tachycardia or incident heart failure [47]; myocardial infarction, atrial fibrillation, congestive heart failure or stroke [48]; new or worsening heart failure, new or worsening arrhythmias or myocardial infarction [54]; new-onset or worsening cardiac arrhythmias, new-onset or worsening congestive heart failure or myocardial infarction [55]; acute pulmonary edema, new onset cardiac arrhythmia, exacerbation of a preexisting arrhythmia, or myocardial infarction [56]; acute myocardial infarction, acute cardiogenic pulmonary edema, new arrhythmia, acute worsening of a long-term arrhythmia, cerebrovascular accident or pulmonary embolism [57];cardiovascular death, non-fatal myocardial infarction or stroke [58]; non-ST elevation myocardial infarction or ST elevation myocardial infarction, stroke, new episode of atrial fibrillation or deep venous thrombosis and/or pulmonary embolism, new or worsening HF or cardiovascular death [61] new onset or worsening arrhythmia, new onset or worsening heart failure or myocardial infarction [63]: new or worsening arrhythmia, heart failure or myocardial ischemia [64].^

^b^ **^Acute coronary syndromes^**^: myocardial infarction [33,37,39,47-49,53-58,60,61,63,64]; unstable angina [44]; acute coronary syndrome [50,51]; acute coronary syndrome or ST segment elevation myocardial infarction [52]^

^c^ **^Stroke:^** ^new-onset neurological deficit [53]; unspecified stroke [52,61]; cerebrovascular accident [57].^

^d^ **^Arrhythmias^**^: incident atrial fibrillation [28,32,33,48-50,52-58,61,64-66]; cardiac dysrhythmias/arrhythmias [34,43]; atrial flutter or fibrillation, and ventricular tachycardia, but excluding terminal arrhythmias [47].^

^e Inpatients without severe vital signs or metabolic abnormalities, altered mental status, suppurative complications or coexisting medical conditions requiring hospitalization [33]; inpatients who survived the first 48 h of hospitalization [41], inpatients not initially admitted to the intensive care unit [42,45]; inpatients with pneumonia severity index (PSI) risk classes I–II [44].^

^f Inpatients admitted to the intensive care unit (ICU).^

^g For ACS, patients from Musher et al. (2007) [47] were included in Corrales-Medina et al. (2009) [51].^

^h Data available for low-risk or high-risk patients if overall cardiac events are considered.^
